# Ischemic and Metabolic Stroke Can Co-occur in m.3243A>G Carriers: A Case Report

**DOI:** 10.7759/cureus.25705

**Published:** 2022-06-07

**Authors:** Josef Finsterer, Sinda Zarrouk

**Affiliations:** 1 Neurology, Neurology and Neurophysiology Center, Vienna, AUT; 2 Genetics, Core Facility of Genomics, Institit Pasteur de Tunis, Tunis, TUN

**Keywords:** metabolic-stroke, stroke-like-episode, mtdna, m.3243a>g, melas

## Abstract

Stroke-like episodes (SLEs) and their morphological equivalent, stroke-like lesions (SLLs), also termed metabolic stroke, are the hallmark of MELAS. Despite increasing knowledge of the properties of SLEs/SLLs, they are often misinterpreted as ischemic stroke, particularly if both ischemic and metabolic stroke occur in the same patient. The patient is a 56 years-old male with MELAS due to the mtDNA variant m.3243A>G in *MT-TL1* and three previous strokes at ages 43 years, 49 years, and 50 years being interpreted as ischemic, focal seizures, depression, right amblyopia, hypoacusis, hyperuricemia, hepatic steatosis, hyperlipidemia, pre-diabetes, and arterial hypertension. He was admitted due to successive worsening of a pre-existing gait disturbance and confusion. Clinical exam revealed dysarthria, word-finding difficulties, right-left confusion disorder, left visual neglect, ataxia, and pyramidal signs. Cerebral MRI showed T2- and DWI hyperintense lesions in a right occipitotemporal location not confined to a vascular territory and in the left posterior border zone. The right occipitotemporal lesion was initially interpreted as subacute ischemia, which is why acetyl-salicylic acid was replaced by clopidogrel. However, after revision of the MRI images, the right occipitotemporal lesion was re-classified as SLL. Arguments for an SLL (metabolic stroke) were the successive onset and the distribution of the lesion. The patient recovered partially from his initial deficits within eight weeks. In summary, SLLs can co-occur with ischemic stroke in the same MELAS patient. Further efforts are needed to clearly differentiate metabolic from ischemic stroke in MELAS patients.

## Introduction

Stroke-like episodes (SLEs), also known as metabolic stroke, are the clinical hallmark of mitochondrial encephalopathy, lactic acidosis, and SLEs (MELAS) syndrome but can occasionally be present in another primary (genetic) syndromic or non-syndromic mitochondrial disorders (MIDs) and even secondary MIDs [1]. The clinical presentation of an SLE depends on the location of the underlying structural cerebral lesion, also known as a stroke-like lesion (SLL), but most commonly presents as headache, hemiparesis, hemianopia, aphasia, apraxia, confusion, disorientation, ataxia, seizures, and vomiting [1]. SLLs present with a distinct pattern on multimodal cerebral magnetic resonance imaging (MRI). SLLs are hyperintense on T2/fluid, attenuated, inversion recovery (FLAIR), diffusion-weighted imaging (DWI), and perfusion-weighted imaging (PWI), hypointense on oxygen-extraction fraction (OEF)-MRI, are not confined to a vascular territory and show dynamic changes over weeks or months [2]. The apparent diffusion coefficient (ADC) maps are heterogeneous, hypo-, hyper-, or iso-intense. Computed tomography (CT) angiography and magnetic resonance angiography (MRA) are usually non-informative [3]. Magnetic resonance spectroscopy (MRS) usually shows a lactate peak within the SLL or even in unaffected cerebral tissue [4]. Fluor-deoxy-glucose (FDG)-positron emission tomography (PET) shows hypometabolism within an SLL. The pathophysiological mechanism underlying SLLs is unknown, but three main hypotheses have been proposed to explain the phenotype, the vascular, epileptogenic, and metabolic hypotheses [2]. The vascular hypothesis is based on the assumption that there is endothelial or vascular smooth muscle cell dysfunction. Arguments against the vascular hypothesis are that SLLs are not confined to a vascular territory, that hyperperfusion instead of hypoperfusion occurs within a SLL, that SLLs occur without cardiovascular risk factors, that there is hypometabolism within a SLL, and that MR spectroscopy shows an increased lactate peak within such lesions. Despite increasing knowledge of the clinical and imaging features of SLEs/SLLs and despite knowing of the underlying genetic defect, metabolic strokes are still often misinterpreted as ischemic strokes [5], as in the following case.

## Case presentation

The patient is a 58-year-old male with a history of hypoacusis, previous strokes at age 43y, 49y, and 50y being interpreted as ischemic, recurrent focal seizures, right amblyopia since childhood, hyperuricemia, depression, panic attacks, steatosis hepatis, perioral seborrheic dermatitis, lactic acidosis, hyperlipidemia, and arterial hypertension. Previous strokes clinically manifested with flickering of the left eye, lower left quadrant anopia, left-sided ataxia, multimodal memory deficits (could not recall words, images, complex contents), depression, and holocrane headache (first episode), focal myoclonic seizures, right-sided hemiparesis, gait disturbance, memory deficits, and dysarthria (second episode), and weakness of the left leg and seizures (third episode). Genetic processing at the age 50 years revealed the mtDNA variant m.3243A>G in MT-TL1 with a heteroplasmy rate of 70%. At home he could walk with a rollator. The patient was a non-smoker and a non-alcoholic. The patient’s mother had diabetes, myocardial infarction, carcinoma, and had undergone valve replacement therapy. Recent medications prior to admission included levetiracetam (2000 mg/d), lorazepam (1 mg/d), acetyl-salicylic acid (100 mg/d), ezetimib (10 mg/d), bisoprolol (2.5 mg/d), and escitalopram (5 mg/d).

The patient was admitted at age 56 because of worsening gait disturbance and confusion that began four days prior to admission. No recent seizure had been observed prior to admission. Clinical examination revealed ataxic dysarthria with halting speech, word-finding deficits, right left confusion disorder, visual neglect to the left, pronation in the arm-holding test, hypoacusis, ataxia on the lower limbs, and a positive Babinski sign on the right side. Blood tests showed mild leukocytosis but normal C-reactive protein. A mild hyper-creatine-kinase (CK) emia with a maximum value of 309 U/L (n, <200 U/L) was observed on the first days of admission. The HbA1C value was 6.15 % (n, <6.0 %) and the oral glucose tolerance test was abnormal, leading to the diagnosis of pre-diabetes without requiring medical treatment. The pro-brain natriuretic peptide (BNP) value was 451.6 pg/mL (n, <125 pg/mL). There was mild hypertriglyceridemia. Serum lactate was normal.

**Figure 1:**
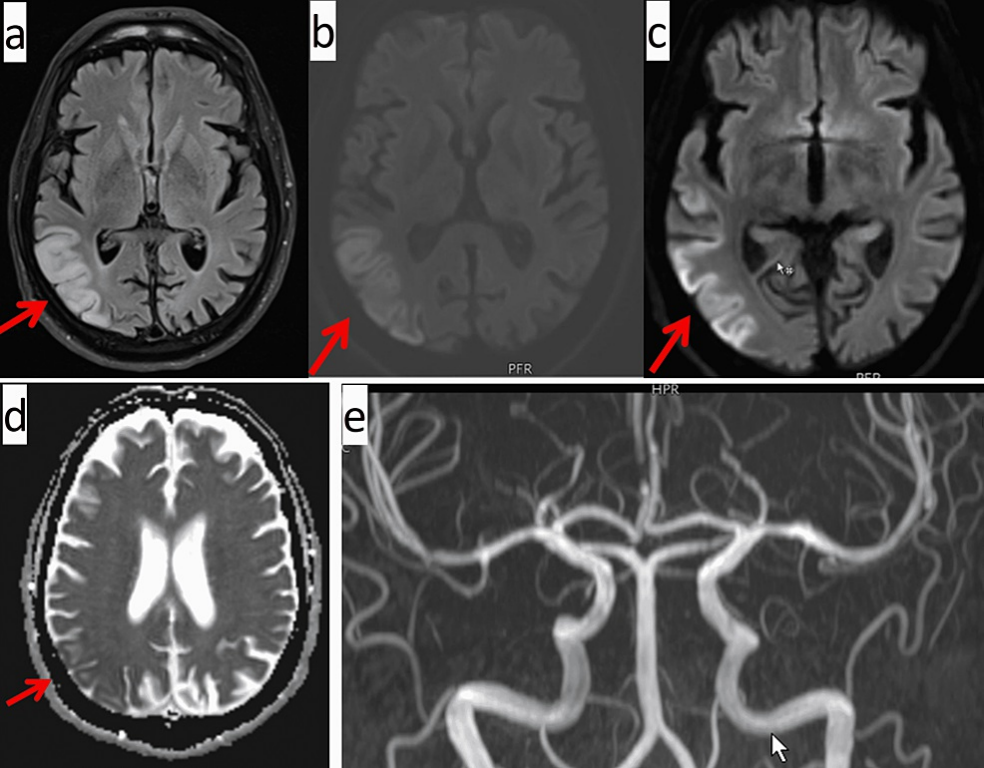
Cerebral MRI on hospital day 2 showing a T-hyperintense lesion in the right occipitotemporal (a), which was hyperintense on DWI (b, c), and slightly hypointense on ADC (d). Additionally, there was a slightly T2- and DWI hyperintense lesion in the left posterior borderzone. MRA was normal (e).

An initial cerebral CT scan revealed lesions in the right temporo-occipital region and the left parieto-occipital region, which were interpreted as post-ischemic. There was bilateral basal ganglia calcification. Cerebral MRI showed a T2- and DWI hyperintense lesion in the right occipitotemporal area, that was slightly hypointense on ADC and a slightly T2- and DWI hyperintense lesion in the left posterior border zone (Figures 1a-1e). MRA revealed no occlusion of any of the large cerebral arteries. The lesions were interpreted as subacute, multi-stage ischemia. Carotid ultrasound showed plaques in the right bifurcation and outflow of the right internal carotid artery, but no significant stenosis or occlusion. Acetyl-salicylic acid was replaced by clopidogrel. A first EEG on the second hospital day showed no epileptiform discharges. On hospital day 10, a series of focal seizures occurred, which is why perampanel was added to the existing anti-seizure drugs. A second EEG on hospital day 12 showed generalized periodic discharges (1c/s) and generalized slowing. The ECG showed neither atrial fibrillation nor any other arrhythmia. Echocardiography was not informative. The further course was characterized by a continuous improvement of the initial gait disturbance and the state of confusion. Despite this development, the patient was transferred to a nursing home.

## Discussion

The patient is interesting because of MELAS with co-occurrence of an SLE and ischemic stroke. Reasons for the misinterpretation of the SLL as ischemic were that the patient had a mild cardiovascular risk factor profile, that previous neurological events had been interpreted and treated as ischemic strokes, and that some of the MRI features were indicative of an ischemic lesion. Imaging findings that argue in favor of ischemia are the mild hyperintensity on DWI and the mild hypointensity on ADC. The cardiovascular risk factor profile included arterial hypertension and hyperlipidemia. However, blood pressure was well controlled and normal throughout hospitalization. Triglycerides were slightly elevated despite medication with ezetimibe. Arguments against the interpretation of the right occipitotemporal lesion as an ischemic stroke in the index case are that the clinical appearance did not correspond to the definition of a stroke (“acute onset neurological deficit”), that the lesion could not be assigned to a vascular territory, that the cardiovascular risk factor profile was well controlled, and that the MRA showed no stenosis or occlusion of a major cerebral artery. Since the left posterior border-zone lesion was interpreted as a subacute ischemic stroke, it is understandable that acetyl-salicylic acid was replaced with clopidogrel.

Although an ischemic stroke in the right occipitotemporal area cannot be entirely ruled out in the index patient, there are more arguments in favor of metabolic than ischemic stroke. Strong arguments for an SLL (metabolic stroke) are the clinical presentation with subacute onset of gait disturbance and confusional state, the diagnosis of MELAS, and that the lesion was not consistent with a vascular territory. Investigations that could further support the diagnosis are PWI sequences, OEF sequences, FDG-PET, MRS, and FDG-PET. In the case of metabolic stroke, there is a mismatch between hyperperfusion on PWI and hypometabolism on FDG-PET within an SLL, as shown in previous cases [6]. Although detailed imaging results of the last three strokes were no longer fully available, it cannot be ruled out that these events were actually SLEs.

Whether seizure activity triggered the development of the SLE, particularly the neuropsychological deficits at onset, remains speculative but is conceivable since SLEs are frequently accompanied by seizures [7]. Arguments against a seizure as the cause of the lesion are that relatives or caregivers did not observe a seizure shortly before or during admission, that the initial EEG did not show any epileptiform discharges, and that the lesion did not show an increase in volume, as is often found in seizure MRIs [8]. Another argument against seizures as the trigger of the SLL is that the patient suffered a series of left focal seizures not earlier than on the 10th day of hospitalization.

## Conclusions

This case demonstrates that ischemic and metabolic stroke (SLEs/SLLs) can co-occur in MELAS and that therefore metabolic stroke can be misinterpreted as ischemic stroke. Multimodal MRI and FDG-PET can help to delineate both entities. The simultaneous occurrence of metabolic and ischemic stroke in an m.3243A>G carrier requires treatment of both conditions. Further efforts are required to unequivocally delineate metabolic from ischemic stroke in MELAS patients.
